# Understanding the Sexual and Reproductive Health Experiences of Refugee and Host Community Adolescents and Youth in Rwanda During COVID-19: Needs, Barriers, and Opportunities

**DOI:** 10.3389/frph.2022.799699

**Published:** 2022-03-28

**Authors:** Katherine Meyer, Monique Abimpaye, Jean de Dieu Harerimana, Christina Williams, Meghan C. Gallagher

**Affiliations:** ^1^Save the Children USA, Department of Global Health, Washington, DC, United States; ^2^Save the Children International, Rwanda-Burundi Country Office, Kigali, Rwanda

**Keywords:** adolescent, youth, COVID-19, sexual, reproductive, health, humanitarian, refugee

## Abstract

**Background:**

COVID-19 has exacerbated the sexual and reproductive health (SRH) needs of those affected by humanitarian emergencies, particularly affecting adolescents and youth, whose needs are often neglected during crises. In Rwanda, the situation for refugees in Mahama Refugee Camp has worsened, as COVID-19 lockdown measures have increased needs while restricting access to basic services. Few assessments have been conducted on the SRH needs of refugees in Mahama camp, including adolescents and youth, since COVID-19. To address this gap, Save the Children (SC) undertook research utilizing SenseMaker to collect data on the SRH needs of adolescents and youth in Mahama camp, as well as in the surrounding host community.

**Methodology:**

SC used SenseMaker to collect 745 data entries from adolescents and youth in Mahama camp and the surrounding host community. The application was pretested with adolescents and youth in Mahama camp before initiating the research. SenseMaker asks participants to share their stories in response to a prompt; our prompt asked participants to describe their experience seeking help with their health during COVID-19. The research team analyzed the data using simultaneous coding to examine key themes. The results were discussed with SC staff to validate the coding analysis results before conducting four focus group discussions to further clarify results and propose action steps in response to the findings.

**Results:**

Many adolescents and youth reported significant difficulties accessing SRH information and services, including stigmatization among service providers. Provider biases and negative attitudes were repeatedly cited as barriers. Stories collected during COVID-19 show how these biases and judgmental attitudes continue to adversely affect access and use of SRH services for young people. Coercive, non-consensual, and transactional sexual incidents were reported from adolescents and youth. They cited reduced time in education spaces as a source of distress as well as increasing their level of sexual activity and associated risks. Limited data exists for SRH needs among adolescents and youth during COVID-19 in humanitarian settings. This study adds to the evidence, making the case for increased SRH prioritization for adolescents and youth in humanitarian settings, particularly when facing overlapping crises like during the COVID-19 pandemic.

## Introduction

Approximately half of the population living in crisis-affected contexts are younger than 20 years old (1). Adolescents—individuals aged 10 to 19 years old—and children who age into adolescence during a crisis are significantly affected by humanitarian emergencies. As humanitarian emergencies have become more protracted, adolescents can remain refugees, displaced, or in need of aid for up to 20 years, comprising all or most of their lives and impacting their educational, economic, and health outcomes (2). During crises, adolescents' health needs, particularly their sexual and reproductive health (SRH) needs, increase dramatically as they are exposed to many risks and conditions for which they are not prepared (3, 4). Family and social structures are disrupted, which could include a sudden loss of resources and the protection of family and friends, heightened insecurity, and a collapse of law and order (5). These conditions hinder adolescents' access to SRH services and information, while increasing risks to sexual violence, abuse, and exploitation; sexually transmitted infections (STIs); unintended pregnancies; unsafe abortions; child, early, and forced marriages (CEFM); transactional or survival sex; and forced recruitment into armed forces (5). Pregnancy and childbirth are particularly dangerous for adolescents due to their lack of biological maturity, putting them at higher risk for complications, morbidity, and mortality (6). Despite the known SRH risks and needs among adolescents and youth, particularly adolescents and youth living in humanitarian contexts, the services provided are often inadequate or fail to deliver the intended impact (7). Health interventions targeting adolescents often categorize them with children or with older youth, neglecting their unique needs and preferences (7).

In addition, humanitarian emergencies often face compounding crises. The Coronavirus pandemic (COVID-19) thrust many communities into a crisis and exacerbated already dire conditions in emergency contexts. At the start of the pandemic, researchers estimated that even a 10% decline in provision of SRH services due to COVID-19 would have a drastic effect on the health of women and families in low- and middle-income countries, including an uptick in the number of women with an unmet need for modern contraception and higher numbers of unintended pregnancies per year (8). Lockdown measures as a result of COVID-19 disrupted supply chains for contraceptives and restricted travel to health facilities, while economic declines related to the pandemic increased gender-based violence (GBV), CEFM, and other rights violations—all further intensifying SRH needs for adolescents (9).

Rwanda, a sub-Saharan African country, was not spared from the effects of the pandemic. After the first case of COVID-19 was reported in March 2020, the Government of Rwanda initiated lockdown measures to stop the spread of the virus, including closing schools (10). While Rwanda continues to report low numbers of COVID-19-related deaths due to quick initiation of previously developed preparedness strategies, the effects of the pandemic have had tremendous impacts upon refugee populations living in the country. Refugees faced shortages of food, loss of livelihoods and employment opportunities, and limited access to health services—leaving many refugees without the means to address their basic needs (11, 12). Humanitarian organizations reported difficulties accessing refugee camps due to lockdown measures and low uptake of services, including SRH services (13, 14).

More than 127,000 refugees and asylum seekers from Burundi and the Democratic Republic of the Congo were living in Rwanda as of July 2021 (15). From that population, ~48,000 refugees and asylum seekers were living in Mahama refugee camp, or nearly 38% of the refugee and asylum seeker population (16). More than half of the camp's population is under 18 years of age (16). For adolescent refugees in Rwanda, the situation largely mirrors vulnerabilities found in other humanitarian settings—with adolescents facing significant child protection and health risks (17–20). Agencies reported a lack of funding for cash, livelihoods, education, health, and other initiatives for adolescents and youth, particularly with so many young people out of school during lockdown measures and limited access to radio, internet, and other equipment to utilize online learning opportunities (21). However, less is known about how COVID-19 affected access and quality of SRH services for adolescents and youth living in refugee settlements.

While research has been conducted on the SRH needs of adolescents and youth in emergency situations, few have examined the impact of COVID-19 upon refugee adolescents and youth. Though there have been calls to ensure the continued delivery of SRH services in the face of the COVID-19 crisis, adolescents in sub-Saharan Africa have been often neglected from COVID-19-specific policy considerations (22–24). Limited studies have looked beyond gender differences to understand the diverging needs of adolescents, particularly in emergencies, and the related programmatic adjustments required to ensure the services are appropriate for all adolescents (7). Of the studies that have looked at the SRH needs of adolescent refugees in Rwanda, many have focused on sexual violence, abuse, and exploitation among adolescent girls, and did not examine adolescents' experiences based on different characteristics of the adolescents, such as age groups, gender identities, and others (17–20).

With support from the Government of Netherlands Ministry of Foreign Affairs, Save the Children (SC)—in coordination with partners from the Inter-Agency Working Group on Reproductive Health in Crises (IAWG)—conducted a research study examining the experience of adolescents and youth accessing health services during COVID-19 in Mahama camp and the surrounding host community. This study did not examine conditions prior to COVID-19. The aim of the study was to provide information on the experiences of adolescents and youth who were seeking health services during COVID-19. The study was conducted at the height of the COVID-19 response, from September to November 2020. The study collected more than 700 entries from adolescents and youth, asking them to describe their experience accessing health services in the last 3 months. The study disaggregated the stories by several characteristics (e.g., age, gender, ability, etc.). By looking at a wide range of adolescents and youth both in the number of stories collected and diversity of ages, genders, and other factors, this study bolsters the limited body of evidence advocating for further prioritization of SRH services and information for young people in emergency settings, even amid compounding crises like COVID-19.

## Materials and Methods

### Study Design and Data Collection

The researchers hypothesized that SRH information and services delivered during the COVID-19 crisis were not meeting the needs of adolescents and youth in Mahama refugee camp and the surrounding host community. In order to understand the reasons why and make some recommendations, the SenseMaker® approach was utilized to collect, describe, visualize, and make sense of the data. This approach asks participants to share their particular story or experience in response to a prompt. Due to sensitivities surrounding SRH within Rwanda, particularly with discussing SRH with adolescents, and the potential lack of information or understanding about SRH among adolescents and youth, the research team chose to phrase the prompt in terms of broader health services, instead of specifically asking about SRH services. The prompt was:

*If you have needed to seek help with your health since COVID-19 has started, please describe something from this experience which stands out for you. If you needed professional health care during COVID-19, but could not reach it, let us know what you did instead*.

SenseMaker asked respondents to answer a story prompt *via* a written (typed) or audio (recorded) response. Participants chose where they wanted to record their story to ensure auditory and visual privacy and confidentiality. To answer questions about their story and demographic questions, the enumerators asked these questions in a private location of the camp compound, where no other people could hear or see what they were discussing. Regarding their stories, participants were asked a series of questions using “modulators” of experience, or themes that might influence the experiences of adolescents and youth. These modulators involved showing participants geometric shapes containing concepts and words designed to reflect potential themes of their story shared. After answering the questions about their stories, respondents answered a short survey of demographic questions for researchers to understand the composition of the target audience.

The research team collected data *via* a project web site, accessible *via* a secure tablet application. All data from the project is available only to a limited number of staff on the research team *via* SenseMaker's web site and software. The data will not be accessible after 3 years. Local enumerators were trained to collect stories from participants using the application. Participants were asked to share a story about their experience seeking health services since the start of the COVID-19 pandemic. After sharing the story, participants were asked questions using modulators of experience, as well as demographic and survey questions, from a developed guide. Participant stories were transcribed and translated from Kinyarwanda into English by SC before being uploaded onto SenseMaker's software.

After collecting all of the stories, the research team conducted four focus group discussions with four population groups, including adolescents and youth (one for adolescent and youth girls, one for adolescent and youth boys), health providers, and community members and parents. These focus group discussions helped clarify and validate results from the stories, as well as provide a space for adolescents and youth, as well as other stakeholders, to respond to proposed recommendations from the research team. The focus group discussions were tailored for each audience; however, all followed a similar structure beginning with questions about the current situation for adolescents and youth during COVID-19, reactions to emerging themes from the adolescent and youth stories, and ending with reactions to proposed recommendations from the research team.

### Study Setting

The study took place in Mahama Camp and the surrounding host community in Kirehe District, located in Eastern Province, Rwanda. The district sits in the far south-eastern corner of Rwanda and borders Tanzania and Burundi. At the time of the study, the majority of the refugees in Mahama camp were from Burundi.

### Study Population and Sampling

SC collected data from adolescents (10–19 years old) and youth (20–24 years old) in the two location sites using cluster sampling outreach methods. The majority of entries collected were from adolescents and youth living in Mahama camp (77%) compared to those living in Kirehe District (22%) and elsewhere in Rwanda (1%, included responses marked “other”). Only respondents aged 10 to 24 years old were included in this study. The research team collected data from 745 entries from adolescents and youth. We chose to target adolescent girls in higher numbers as they are more affected by access constraints to SRH services during emergencies. The composition of respondents' characteristics is included in Table 1.

**Table 1:** Study population and sampling.

**Age**	**Gender**	**Education status**	**Marital status**	**Ability characteristics**	**Pregnancy status**
Adolescents aged 10–14 years old (14%)	Female (75%)	Not completed secondary (54%)	Not married (86%)	Difficulty with memory (21%)	Not pregnant at time of survey (87%)
Adolescents aged 15–19 years old (61%)	Male (24%)	Not completed primary (26%)	Married (10%)	Difficulty with sight (15%)	Pregnant at time of survey (13%)
Youth aged 20–24 years old (23%)	Non-binary (1%)	Completed primary (10%)	Widowed (1%)	Difficulty with mobility (6%)	
Young people aged 25 or older (1%)	Prefer not to say (<1%)	Completed secondary (4%)	Divorced or separated (1%)	Difficulty with hearing (5%)	
Prefer not to say (1%)		Did not complete any education (3%)	Prefer not to say (2%)	Difficulty with self-care (4%)	
		Completed other education (3%)			

From the 745 entries collected, 112 entries provided no data or incomplete data, resulting in 633 entries available for analysis. The research team screened those 633 stories, and analyzed a total of 517 stories, as 116 entries were either duplicate entries, or did not pertain to accessing health services.

Focus group discussion population and sampling: The focus group discussions comprised four groups of respondents, including a sample of adolescents and youth refugees from Mahama camp, separated by sex; a sample of community members residing in the camp (opinion leaders and parents); and a sample of health providers working in the camp.

### Data Analysis

The responses related to experiences accessing health services from the focus group discussions with adolescents, youth, community members, parents, and health providers were included in the Dedoose analysis with the 517 SenseMaker stories and were similarly incorporated into the results' emerging themes sections. To analyze the transcripts from SenseMaker and the focus group discussions, a directed content analysis approach was applied and preliminary codebook was developed using an iterative coding process. Key themes were identified using line-by-line open coding within Dedoose using theoretical memo writing throughout in order to connect concepts.

The transcripts were first read by two researchers to identify overarching themes for the creation of draft codebooks. The researchers worked in tandem to develop the final codebook. The codebook was organized by general themes and sub-themes. Eight transcripts were coded separately by the two researchers using the draft codebooks and the results were discussed to revise the codebooks, adding, deleting, or collapsing codes as necessary.

Once codebooks were finalized, coding was performed in Dedoose independently by two researchers; selected transcripts were coded by a third researcher to ensure reliability and validity of the coding. The consistency of coding was assessed by inter-coder reliability, calculated as the number of agreements divided by the total number of agreements and disagreements. Disagreements were discussed and resolved until the inter-rater agreement was 90% or greater. Finally, the data were analyzed and presented using the words of the respondents. The results were discussed with SC's Mahama refugee staff, including enumerators from the study, to validate the coding analysis results. Following those discussions, SC conducted four focus group discussions to further clarify results and begin discussing proposed action steps for SC and other health actors in the camp to take in response to the findings. The proposed action steps included reviewing COVID-19 protocols related to triage categories and treatment prioritization, training staff on providing SRH information and services to adolescents and youth, installing a permanent staff member dedicated to youth at the youth center and health center, restarting and/or adapting youth programming in the camp to accommodate COVID-19 protocols, co-developing communication materials with adolescents, youth, and community members, and engaging other community members in health messaging and service provision. Feedback from the focus group discussions related to the proposed action steps is included in the Discussion section.

As this was a qualitative study, the themes included in this paper do not represent the experiences of all adolescents and youth in the study setting, nor did all of the themes included below appear in every story collected. The themes are an aggregated summary of their shared experiences according to the SenseMaker methodology employed as described in the above text.

### Ethical Consideration and Approval

Prior to data collection, the research team sought and received ethical approval from the Rwanda National Ethics Committee (RNEC). The protocol number for the ethics review was No. 958/RNEC/2020. This committee is the leading authority on providing recommendations on the ethical norms in Rwanda. The study was also registered with SC's Ethics Review Committee.

Enumerators collected assent and consent forms from participants, as well as parental consent and adolescent assent for adolescents under 18 years of age. To ensure that the research maintains the highest rigor and complies with ethical standards, data collection was completed by 24 data collectors from SC's data collection database; these collectors have all been trained in using the data collection tools. Privacy and confidentiality of the interviews was ensured, as outlined in the Data Collection section. The 24 data collectors were divided into four teams, comprised of one team leader and five data collectors. For oversight, SC's project midwife officer and research coordinator acted as supervisors during data collection, and its head of research, evaluation and learning was in charge of the overall survey. In advance of collecting data, enumerators explained the purpose of the study to obtain assent and consent for the research and participation in the study. Each participant received an explanation on the purpose of this survey and was requested to fill a consent/assent form prior to their participation in the study (no one was interviewed without their informed consent/assent). The consent/assent form was read and the participant was given time to ask questions. Each was given a copy of the consent form to keep. The recordings and all data collected are only accessible *via* SenseMaker's web site, using password-encrypted software that is provided to a limited number of the research team. The data will be destroyed after three years.

**Assent of adolescents under 18:** Each adolescent who is under 18 assented to be part of the study and parents or legal caregivers signed the consent form on behalf of the child.

## Results

From analyzing 517 stories with adolescents and youth, several themes emerged, which were categorized into four areas of emerging themes: enablers; barriers and obstacles; outcomes and consequences; and opportunities. Below each theme, several subthemes were identified with related quotes from adolescents and youth to help illustrate the emerging themes. Responses from the focus group discussions—which were employed to provide greater context and, in effect, validate the results—were incorporated within the thematic areas, where relevant. When responses from the focus group discussions differed from the adolescent and youth story responses, additional language was included to provide the nuanced perspectives and responses.

Overall, the majority of adolescents and youth who participated in this study found areas for improvement in the services they received. These results were anticipated as literature shows COVID-19 strained human resources, supply chain infrastructure, and delivery of essential commodities for health systems across the world (25). However, despite these constraints, some adolescents and youth reported positive experiences accessing health services.

Finally, results from the focus group discussions regarding participants' reactions to the research teams' proposed solutions is integrated throughout the Results and Discussion sections. This practice aligns with global best practices regarding meaningful participation of the affected population, which includes adolescents, youth, community members, and health staff. Meaningful participation understands that for successful implementation and longevity of a project, people must be able to participate in the decisions that directly affect them.

### Enablers

For adolescents and youth who described receiving the SRH information and services they requested, they stated that they trusted health staff, received appropriate counseling, and were treated well at the facility. They talked about receiving training outside of the facility from youth programs, including community mobilization activities that persisted during the pandemic. Outside of their experience with the health facility, adolescents and youth talked about how their assets contributed to positive outcomes. They described how having goals, a supportive family and peer network, and employment enabled them to make healthy decisions for their lives.

“I went to the health center and wanted to get tested for HIV/ AIDS because I thought I was infected; they tested me and found no problem. They immediately gave me condoms so that for the next time if I had sex again, I would use it to protect me against any infection because if I was infected [I could be at risk of dying] nothing else could be done except the death. I took condoms and gave them to others and I decided to abstain until I got married instead of being exposed to those infections.” (15–19-year-old Male, Mahama Camp)

“In this period of Corona [COVID-19], I went to the health center to be tested for HIV. They received me warmly, and I am thankful for the service rendered to me.” (15–19-year-old Female, Mahama Camp)

“Peer educators taught me; they taught me about abstinence, patience, and how I can behave against [take measures to avoid] unwanted pregnancies for the sake of my studies.” (15–19-year-old Female, Mahama Camp)

“For me, normally, when it [menstruation] first happened to me, I was not aware. I did not see it elsewhere nor to [in] my elder sisters. Though I was not familiar with such a thing [menstruation], I asked [my] mom and she explained everything and how I can manage and behave. She gave me pads. As a parent, she advised me [on how] to protect myself and [how] to avoid those who [try to] can mistreat me [by lying to me] for they can lie to me and [advised me on how to avoid unplanned pregnancy] find myself being impregnated unwillingly.” (15–19-year-old Female, Mahama Camp)

### Barriers and Obstacles

#### Changes Due to COVID-19

Adolescents and youth discussed how their environment has changed since the COVID-19 pandemic began, including how those changes have impacted their ability to access health services and other youth services. Adolescents and youth discussed how youth programming and health services for adolescents and youth were better before the pandemic, stating that services were higher quality and more timely. Adolescents and youth believed health providers were more welcoming and treated them better prior to the pandemic. Health facilities have implemented new protocols since the pandemic, and some services have been disrupted or discontinued. For example, adolescents and youth believed circumcision services have been reduced considerably and reported being refused services if they did not have a fever or facing considerable delays due to the number of patients being treated for COVID-19. Some adolescents and youth also reported fears of becoming infected with COVID-19 if they went to the facility. Adolescents and youth from focus group discussions reported that they received different medical treatments during COVID-19 for the same illness they had suffered and received treatment for prior to the pandemic.

“…I met a lot of challenges in this COVID-19 period. The way we got service at YFC (youth-friendly center), it seems it is gradually diminishing [The services available at the YFC decreased during COVID-19]. I personally went to the hospital to ask for treatment. I was suffering from those sexually transmitted diseases. I did not find the nurse on that day. The peer educator told me that the nurse is not around. I went back home. Usually, we meet the nurse on Thursday and Friday, but in this days of corona virus, he doesn't come regularly. For me, this is the main challenge I faced in terms of reproductive health. In addition to this, other people may need the help of the nurse on [for] family planning and they miss him [cannot find the nurse/doctor]. This may lead you to [having an] unwanted pregnancy. What I may suggest is that when there is a pandemic that will cause the closure of some services, you may take risks [health actors should take action] to help young people and others because you may probably not be killed by corona and you get killed with other unknown diseases like syphilis, gonorrhea [you may face other risks outside of COVID-19 that require medical attention]. So if there any kind of pandemic, you should take measures to fight against other diseases because they are still there in abundancy [they are still present everywhere], thank you.” (15–19-year-old Female, Mahama Camp)

“In this period of COVID-19, things [have] become harder, in this period that schools are closed [with the closure of schools]. Most of the time, boys come closer [visit us] and lie to us [deceive us into transactional sex], consequently, we got unwanted pregnancy [resulting in unplanned pregnancy]. At the hospital, they do not treat you unless you have fever. We ask from you to give us some materials such as slippers, soaps, and lotion that hinder boys from lying to us and it will be of a great value.” (15–19-year-old Female, Mahama Camp)

Aside from health services, adolescents and youth stated how COVID-19 had affected their lives in other ways—shutting down school or education services, reducing youth programs and activities for SRH, and decreasing their ability to pay for basic items.

“Here at Mahama, services are provided, but it was better before the outbreak of Coronavirus when a lot of people were able to be served at the same time…Before, they used to carry out education campaigns and many people could have access to such information. But now, only one person can be given a service while respecting social distancing guidelines. For example, an educator could handle 50 people in a week, now they can only handle 20 people because of the pandemic. There are even some services that had to be put on hold like circumcision. Many children went to Save the Children to watch movies on reproductive health while others got [received] trained. When you get to watch something on video more [multiple] times, one [you remember] remembers faster than just hearing. Services are still being provided but they do not reach many people in a short time. [For] Other services related to reproductive health, you might ask someone the age when their bodies start to experience changes [people seeking SRH and sexuality education information] and get no answer because they have no information [cannot get their questions answered or have access to find the SRH information they are seeking].” (15–19-year-old Male, Mahama Camp).

#### Inadequate Health Facility Services

The research team saw repeated mentions of unsatisfactory facility conditions and inadequate service quality experiences described by adolescents and youth. Adolescents and youth reported shortages of health staff and unavailability of health staff, including service providers, nurses, and youth-dedicated staff. Adolescents and youth discussed increased risk of unprotected sex, unplanned pregnancy, drug use, and STIs, including human immunodeficiency virus (HIV). Adolescents and youth cited COVID-19 as a contributing factor to these increased risks, but also noted how changing conditions, such as inadequate service provision and treatment, had contributed to these health concerns. Parents and community members overall did not feel that quality of service circumstances had changed for youth since the pandemic. In their opinion, the only aspect that had changed for adolescents and youth was a decrease in youth programs. Parents then admitted that there had been a reduction in staff at health facilities (adults themselves also faced issues with triage and doctors only seeing patients with a high fever) but claimed that it had not stopped how health providers received children and youth. Parents and the community believed that youth were able to receive the services they needed due to support from peer educators.

Adolescents and youth talked about delays to health services, including delays in receiving care and long wait times to receive services. Adolescents and youth believed these human resource gaps were due to COVID-19 as they discussed the camp only allowing a certain number of providers during the pandemic and reduced number of days that health staff were able to see adolescents and youth. In some cases, the extent of the delays faced by adolescents and youth resulted in unplanned pregnancies, continued pain as a result of STIs or other ailments, and self-administered removal of contraception when health staff were unable to provide timely services to them. They believed health staff did not have the time to treat adolescents and youth, with health staff stating they were “too busy” or had too many other patients to examine or provide services to adolescents and youth. Health providers also confirmed the adolescents' perspectives of a shortage in health staff due to COVID-19 and that since the pandemic started, providers do triage patients with severe cases and some services, especially SRH services such as circumcision, have stopped.

“Before COVID-19 came, I could take my tablets regularly but when COVID-19 came, I went to pick medicine, where I usually get them [obtain the tablets], and found that the doctor did not come. So I skipped taking the tablets and conceived an unwanted pregnancy.” (10–14-year-old Female, Mahama Camp)

“On [In] my story that I want to share with you, I got [had an] unplanned pregnancy. I even went to see the nurse and did not see him/her. S/he had given me the appointment before COVID-19. When I went there (COVID-19 had started), I missed them. That is why [how] I found myself [became] pregnant. You understand how life is hard. It reached the time I even got [I also contracted STDs] STDs and I kept looking for them [health staff] and did not find them [health staff]. It is not only that. The one [person] who impregnated me refused the responsibilities because he knew I was under [taking] family planning [contraception]. That is my story.” (15–19-year-old Female, Mahama Camp)

Adolescents and youth reported unfriendly demeanor or treatment from health staff, including health staff not making adolescents and youth feel welcome at the facility and exhibiting judgmental attitudes or behavior toward adolescents and youth. Adolescents and youth emphasized the power dynamics they have felt when interacting with health staff and the lack of agency they have felt when asking for what they need or complaining about services. One respondent from the adolescent and youth boy focus group discussion stated “you never complain with someone who has power over you.” In the focus group discussion with health providers, they talked about the difficulties working with youth and adolescents, and receiving confusing messages about their reasons for coming in for a consultation.

“Working with youth and adolescents is not easy. They can come and told [tell] you the[ir] problem they have, but when they meet the doctor, they tell the different stories. When they cannot get what she/he wanted [wants], they tell everyone they meet that she/he got a bad service.” (Participant from health provider focus group discussion)

Adolescents and youth stated that health staff asked many questions to adolescents and youth, making them feel anxious. Health staff told adolescents and youth to be patient with their situation and showed a lack of empathy or compassion for the adolescents' and youth's concerns or problems, according to adolescent and youth stories.

“…I went to the hospital. The first time I did not see them [health staff] and went back [to the hospital]. The second time I saw them [health staff] but what annoyed me, after narrating how I feel, s/he told me to be patient, that God will cure me. It reached the time [After this,] I had sex with a boy and later I heard that he is HIV positive. When I went to look for help, they [health staff] asked me to go and come back with him [the boy I had sex with]. Reaching to him [I contacted him, but] he refused. Coming back to the hospital, they [health staff] did not help me, they insisted for me to come with him.” (15–19-year-old Female, Mahama Camp)

“It was in December when I went to the health center for a family planning service. When I got there, [a health provider] injected me [with a contraceptive method] and I returned home. After COVID-19 began, I went back to the health center, and they refused to accept me because I had no fever. They only accepted patients with high fever, and I was refused to enter. For another time, I went back to the health center and they welcomed me but in a non-pleasant way. They looked at me as if I was invisible, I returned home, and God healed me. I want to say that whoever goes there [to the same health center] with the same family planning problem, we want you [non-governmental organizations] to advocate for us [adolescents and youth] to be [for health staff to be] as welcoming as we want them to be, just as we used to be expecting them to be kind to us but not yet.” (20–24-year-old Female, Mahama Camp)

“Of course during those periods [during COVID-19 lockdowns], there was some change, such as you could come here to the health facility and found that the doctor/nurse isn't around [were unable to find a doctor]. Or when you go to the hospital, you find female parents and you are afraid to express the concern/issue that you have. It was hard and you were afraid saying that if I cross to that door [if I enter the facility for specific service], that person we live together in the community [a person from the community will see me and tell others why I was there] will say that I went there to look for the service X and Y, and it will cause you problems.” (Participant from adolescent/youth girl focus group discussion)

Some stories discussed health providers refusing treatment to adolescents and youth due to judgmental attitudes or bias from health staff and/or health facility policies, including the requirement of the patient's partner to be present to receive tests for pregnancy or STIs, and for receiving contraception, including emergency contraception. While adolescent and youth stories discussed this policy being newly enforced during COVID-19, adolescents and youth from focus group discussions stated that this was an issue prior to the pandemic. Parents and community members also confirmed that health staff were enforcing the partner presence policy with adolescents and youth. When health providers were questioned about the adolescent perspective of the adolescent or youth having to bring their partner when accessing health services, there were mixed responses. Some providers confirmed the need for a partner's presence in order to access services, while other providers stated that in some cases, community health workers (CHWs) were allowed to come with the adolescent to explain why they do not have a partner there so that the adolescent could receive the service they need. Some of the statements by community members and parents were non-confirmatory and somewhat judgmental of adolescents. For example, one adult community member stated in regards to adolescent girls and boys expressing lack of access to services, the community member believed that no one denied them services or was mean to them and that “children are lying” to hide pregnancy from parents and community.

“I had unprotected sexual intercourse, then I went to the hospital to meet the youth doctor. I met [encountered] a challenge because there is a rule that you must go with your [sexual] partner while s/he can refuse or don't be available to go together [or you will be refused service by health providers]. It is a barrier for us. Before COVID-19, it was not like that. I do not know if it is related to COVID-19 or just new rules. We are not comfortable with it, [with health providers requesting sexual partners to accompany the adolescent client to receive services] asking to come with a partner to get such service. Which [This policy] can lead to the disease and the unplanned pregnancies [adolescents and youth contracting STDs and having unplanned pregnancies] because you [adolescents and youth] did not get emergency [receive emergency contraception] within 72 hours.” (15–19-year-old Female, Mahama Camp)

Adolescents and youth from focus group discussions also discussed the fear of imprisonment if they admitted to having sex with a minor or being forced to provide the name of the person who had sex with them if they were a minor and their sexual partner was not a minor.

“…The way [after] you have unprotected sex…you go to see the doctor/nurse telling him/her [and tell them] your problem. [You say,] I had an unprotected sex with my partner, kindly give me those drugs that you take within 72 hours to prevent you from getting pregnant. The doctor/nurse told me to go and bring the person we did sleep together [that I slept with]. You think about bringing the person and you are under 18 years old, if I bring [and think about bringing] the person, the doctor/nurse will write a dossier and they will come and look for him to imprison him [if you are under 18 years old, you have to think about whether or not you want to bring the person you had sex with because if they are 18 years or older and you bring them to the facility, the doctor will make a record of the incident]. So you just give away [So, you do not bring the person or get the services you need].” (Participant from adolescent/youth girl focus group discussion)

Adolescents and youth reported receiving the poor treatment or medicines that did not address their health needs. This includes adolescents and youth not receiving the appropriate treatment, information, or supplies for their requested services, such as not receiving condoms or not receiving contraception. Adolescents and youth also discussed receiving the wrong treatment or poor treatment from providers or the facility staff, resulting in negative health outcomes such as continued pain, unplanned pregnancy, untreated STIs, and determination not to return to the health facility.

“I went to the doctor. I was shaking with a fever. Three days after, I got [went for] a check-up and was given medication to take with me home [home with me]. After taking it, I had a miscarriage and was bleeding. While at the hospital, I spent 2 days [there] without seeing the doctor. I felt disappointed and chose to go back home because I did not receive the kind of service I needed. They came to take me home before I healed. I bled for 2 weeks. I healed from home. Our neighbors were the ones that gave me medicine.” (15–19-year-old Female, Mahama Camp)

“I had a problem of pain during my period. I went to health center, and they denied to help me saying that I had no fever. Then, I decided to back home and patiently [be patient]. I spent the period suffering from that pain at home and finally it ended.” (15–19-year-old Female, Mahama Camp)

“I went for family planning service and they put me on implant. Later, it [the implant caused pain] gave me hard times. I could [was] always bleed[ing] and lost weight. I decided to go back and ask the nurse to remove it, but they refused. It reached the time, and I decided to remove it myself. I was about [almost] dying. I will never go for family planning from what I saw.” (20–24-year-old Female, Mahama Camp)

Adolescents and youth discussed a lack of supplies, including SRH commodities and COVID-19 supplies. In some cases, the lack of COVID-19 testing supplies prevented health staff from being able to examine and/or treat adolescents and youth for other health services. Adolescents and youth also discussed not receiving enough information on SRH to make appropriate decisions for themselves.

“I went to the health center to get tested for sexual infections and I got all the other services I needed, but I did not get tested for HIV because the testing kits were not available yet.” (20–24-year-old Male, Mahama Camp)

“In this time of COVID-19, things are really hard for me. I went [to the health center] for a pregnancy test, [and] I arrived late and found others already in [line]. They refused [let] me to enter unless I got COVID-19 test. At that time, the doctor who was in charge of testing [for] COVID-19 was not around. I waited for a long time under the sunshine from morning to noon; it was very sunny. When I got hungry, I chose to go back home. I did not get any service that day. The next time, I came back [and] they insulted me and asked me why I did not follow the timetable they gave me. In short, it is really hard for us. I wonder if you could add more nurses/ doctors or see what else you could do.” (20–24-year-old Female, Mahama Camp)

#### Disruption to Social Support Systems and Services

Disruption to social support systems and services included discontinuation of school or education sessions and a lack of youth programs or activities due to reduced hours or staffing from non-governmental organizations. These disruptions have led to adolescents and youth spending more time at home and without access to youth services, including community mobilization activities, drama clubs, games, and other social activities. Adolescents and youth talked about how this lack of activities has increased anxiety and led to more risky behaviors, including unprotected sex. Adolescents and youth also talked about increased risks of transactional sex and drug use.

“In this COVID-19 period, it has been very difficult for us because many services have been stopped, [and] we no longer see the nurses at the Youth Friendly Spaces (YFS). We no longer receive the reproductive health education, [and] the certification of reproductive health trainings has stopped. In addition, there is a scarcity of condoms.” (20–24-year-old Male, Mahama Camp)

“In this period of COVID-19, we are meeting different challenges related to reproductive health. There are some services we are not getting as usual. We used to go to YFS (youth-friendly space) for entertainment but we no longer go there. Since we are living at home by ourselves, we need girls to visit us but we are not getting enough condoms. You might have unprotected sex and end up being [contracting] infected with the HIV/AIDs virus. Since the nurse in charge of testing HIV AIDS is [only coming in once a week] coming once in a week, in most cases you do not find him/her in the health center [have difficulty finding a nurse to receive testing services]. For example, I had unprotected sex and went to see the nurse but I did not manage to see her because she was not around.” (20–24-year-old Male, Mahama Camp)

Adolescents and youth discussed a lack of employment and livelihood opportunities, resulting in increased pressures on households to meet basic needs, including money for soap, menstrual supplies, and clothes. Some adolescents and youth reported that a lack of finances to pay for these items had resulted in negative coping mechanisms, such as transactional sex in exchange for money or the needed items. Many of the adolescents and youth who spoke personally about transactional sex or about others engaging in transactional sex stated this topic as an issue for girls. However, during focus group discussions with adolescents and youth, they revealed that this is also a major concern for adolescent and youth boys. Other adolescents and youth discussed dropping out of school to find employment or due to unplanned pregnancy, in addition to increased incidence of CEFM, and the refusal of one's sexual partner to assist the mother with pregnancy, childbirth, or child rearing.

“The support that you can give me, well, I engaged into sexual relations without thinking about it and was impregnated [became pregnant]. I really need help because I am now out of my mind that I could commit suicide if it was possible…Someone whom I slept with denied it. I am really requesting for your help because myself engaged in that in order to sustain my life [I need help to stay alive], ignoring the fact that I was actually destroying it [even though engaging in transactional sex was how I was trying to stay alive]. I thought that I could get a piece of soap and body lotion, but I found myself with nothing at the end, neither soap nor body lotion. Please, find a way in which you can help me because I am so desperate. I really need to be comforted because I am so depressed.” (20–24-year-old Female, Mahama Camp)

“Hello, we are not happy for the things that are happening to us here in Mahama. Boys tell lies to us that they love us. Some girls accept given the poor living conditions here in this camp since we are not even allowed to go out of the Camp. Many girls stay alone small houses, and boys convince us that they will give us money or buy for us high waist trousers…Eventually, girls are persuaded to sleep with boys. It is said by boys that if you are poor and [you will] be given support, then you give back [provide something in return]. Many girls come from poor backgrounds and when parents harass them, they are forced to marry at an early age despite the fact that some parents enhance [provide] positive parenting. I would like to once again inform you that us [girls] are persuaded by small gifts, such as body lotion and sandals. Thank you and always give us advice since your advice is equivalent to [our] parents' guidance.” (15–19-year-old Female, Mahama Camp)

“Of course in this period of COVID-19, I visited a boy and willingly impregnated me [had consensual sex and became pregnant], later on, he told me that he can't afford marrying me due to his economic status, until now he refused to offer me any support.” (15–19-year-old Female, Mahama Camp)

“Normally if someone wants anything bad to you [wants to do you harm], she will use what you need/don't have. If she tells you that she will be providing everything that you need, once she has provided [items, money, etc. to you] once or twice, it is over. You belong to that someone [that person], and she will do everything she want[s] to do with you. If she buys for you that soap or body lotion, she has you in permanent [she has control over you] and [can force you to do] everything she wants you to do. You do it because she has your life.” (Participant from adolescent/youth boy focus group discussion)

Adolescents and youth talked about general poor conditions that existed prior to COVID-19 and/or were exacerbated by COVID-19, including poverty, insecurity, electrical outages, lack of technological services during lockdown, lack of assets or resources available adolescents and youth, and violence, including intimate partner violence. Some stated they did not have support from family, peers, or community members. Others talked about how they were not informed or lacked the knowledge on how to access services or where to go. These conditions have increased mental health needs for adolescents and youth, among other needs.

“Concerning violence, I have a husband but [and] our relationship is bad. We are not living in good harmony. He sometimes beats me during the night and argues [threatens] to abandon me or send me back to my family. Before the pandemic, our marriage was a blessing, as he treated me well. I have a problem of where to go if it happens abandoning me [he leaves me]. I am requesting for support to raise [to provide] basic needs for my children.” (20–24-year-old Female, Mahama Camp)

“I had unprotected sex and because of the lack of information, I didn't know I could have gone to the health center for emergency contraception. I ended up getting pregnant. Afterward, I went to a youth center, but no nurse was there. Therefore, we urge that a full-time nurse at the youth center could help a lot [be available to help] since we are sometimes afraid and ashamed of going to the health center.” (15–19-year-old Female, Mahama Camp)

#### Perceptions of Social Norms and Stigma Surrounding SRH

Adolescents and youth discussed how social norms and stigma surrounding SRH had affected their ability to access health services during COVID-19. Adolescents and youth talked about the social pressures they faced if they had premarital sex, calling themselves failures or saying they had failed to abstain. Many adolescents and youth cited religious reasons or family pressure for wanting or trying to avoid having sex before marriage. Adolescents and youth believed having premarital sex was giving into temptation and discussed feelings of guilt, behaving badly or immorally, or being weak if they did have sex before marriage. Some stories talked about being afraid to speak up or discuss sex due to societal norms or pressures.

“Thanks for giving me this opportunity to speak out [about] the problem we have. The problem is that schools are closed and promiscuity is becoming too much in girls [increasing among girls]. We request you to advocate for reopening of the schools and we go back to school.” (15–19-year-old Female, Mahama Camp)

“As my case as a concern, I was a student and you see this COVID-19, what happened [is] we kept on [were] waiting that we may [to] return to school but we didn't get it [did not return]. The fact that [Because] we have lovers and we have no other occupation as we always spend the day doing nothing here in camp, we are jobless and [do] not have productive activities and you see schools are closed… Thus we have lovers [and] they invite us to their houses and we visit them, [and] then we do sexual intercourses with them [and] we have no other job, that is the job for us.” (15–19-year-old Female, Mahama Camp)

Adolescents and youth talked about an increase in promiscuity among girls and girls being “tricked” into having sex for different reasons. Some stories discussed more girls and boys having sex because of a lack of activities with closures to school and youth activities. Some stories talked about boys lying to girls or making promises to girls in exchange for sex. Adolescents and youth also discussed using traditional treatment instead of consulting health professionals due to pressure from community or family members. The theme of lying or being tricked was also discussed in focus group discussions with health providers and community members, with respondents talking about adolescents and youth engaging in “bad habits” and that girls are “tricked” by older men and not younger boys as “they have nothing to give them”.

“Before COVID-19, we had access to many services, for instance [such as] attending school regularly. But nowadays, we have no access to services due to this period of COVID-19. Life has really changed because it is not easy for one to receive a required service. Boys also trick us since we are not attending school, so we end up accepting to sleep with them [agreeing to have sex with them]. We hope this will come to end and then [we can] return to school and study well hence ignoring boys' tricks [and ignore the boys' tricks]. This will help us return to normal life. Prior to COVID-19, we had [a] normal life, but today we are having unexpected temptations by visiting boys while we were not intending to [that we did not intend to visit].” (15–19-year-old Female, Mahama Camp)

“During COVID-19, I cannot afford menstrual pads because they are now too expensive and [I] chose to use the traditional method, which causes rashes on skin. In addition, I went through an excessive [painful] menstrual cramp and I had no place to get relief medication.” (15–19-year-old Female, Kirehe)

### Opportunities

#### Youth Integrated and Tailored Programs

Adolescents and youth described several areas of opportunity to improve conditions surrounding their experience accessing health services and information. The first of these opportunities is for health organizations to increase youth integrated and tailored programs. Adolescents and youth have strong positive feelings and experiences engaging with youth programs in the camp. They want adolescent and youth activities and programs restarted and increased to offer SRH health information, as well as services, within the health facility and also in youth-friendly centers and other entry points. Adolescents and youth want services offered closer to where youth can access them.

“I went to the health center to seek a service of getting condoms, they really gave them to me and they gave me a lot, but there were some who didn't get them due to fear of going there. However, in the mobilization activities, the peer educators used to travel all over the community distributing condoms to youth, they usually do it and many young people in our area get a lot of condoms.” (20–24-year-old Male, Mahama Camp)

“I was looking for a condom and I could not find any, consequently, I had unprotected sex. So, I beg to bring the condoms closer to us for instance in boutiques and bars near to us.” (20–24-year-old Male, Mahama Camp)

#### Dedicated Youth Staff

Another opportunity to improve conditions for adolescents and youth is to increase the number of dedicated staff for adolescents and youth. They would like to see more peer educators and/or health staff able to provide services at youth centers or facilities. Adolescents and youth requested a permanent nurse or health provider at the youth-friendly center and would like to see more youth-dedicated/trained staff involved in health facilities and programs to make them feel more welcome. Health providers noted that some youth could not share what they were facing with their parents, but by talking to youth counselors, youth had someone close to their age who could advise them on how to navigate the system. They also confirmed that when youth visit the health center, it is helpful for them to come with a peer educator/youth counselor or a youth-friendly center doctor transfer so that the doctor at the health facility understands the urgency of the youth's case.

“…Before the COVID-19 has started, when you had unprotected sex you would go to the doctor [and be] easily accompanied with peer educator to help you get a doctor quickly and then be hopeful that there is no problem behind [be reassured that nothing is wrong]. But when [since] COVID-19 started, now things are really difficult. As for me, when the COVID-19 started, I had sex and then I asked for help with a peer educator, and we lacked [could not find] a doctor to help me. I returned back in a bad mood with a lot of thoughts and I was worried, due to that anxiety I felt I passed long time [spent a lot of time] in depression. Luckily, for me I found out I was not pregnant, I felt happy, but before I was really worried. We wish you would do something to make a difference in this COVID-19 period. I wonder why the doctors are no longer available. I don't know if it is there scarcity. Help us get it back as before. Thank you very much.” (20–24-year-old Female, Mahama Camp)

#### Bolster Social Support Systems and Services

Lastly, adolescents and youth talked about assets as a critical piece to improving access and use of health services. They identified family and peers, as well as having goals, as protective factors and increasing the likelihood for them to make healthy choices. Adolescents and youth from focus group discussions also highlighted the role of father-educators, as well as other community leaders, in helping adolescents and youth access (and have the confidence to ask for) the medications or treatments they need when health facilities are overcrowded. Adolescents and youth talked about the importance of employment or financial support in making healthy decisions. Having access to basic items can prevent adolescents and youth from needing to resort to negative coping mechanisms, such as transactional sex or dropping out of school.

“During this period of COVID-19, I get different advice from my parents. Of course, there is a lot of temptations but I am luckier to have parents who keep close to me. Therefore, we urge other parents to keep close to their children.” (15–19-year-old Female, Kirehe District)

“…As now [Currently,] we have nothing to do, no [we are not] going to school, [and] we only stay home. We need something to occupy us, like those trainings. Sometimes we try to listen to the radio in other [in order] to get news, but there are times when there is no electricity or when radio batteries ends up [are used up] and you can't get the information you have been needing. Lack of information and poverty make[s] many young girls had [have] an unplanned pregnancies, not because they want to, but [because] they are seduced by things they don't have. If you had information, no one would deceive you and even if it happened to you [you are deceived], you would say [that] at least you knew, but since we don't have anyone to give us the information, many young girls fall into temptation.” (15–19-year-old Female, Mahama Camp)

Health providers and community members from focus group discussions also discussed the importance of father-educators, and community members noted that adolescents can receive advice from many sources, including father-educators, community leaders, youth educators, CHWs, health staff, and their parents. Health providers and community members discussed how parents should be providing SRH and other health information to adolescents and youth but that sometimes parents are afraid to speak to their children about these topics. One community member shared this example, if a girl was to talk to her parents about a potential pregnancy, she would be kicked out and the man would say she was lying.

## Discussion

This study aimed to examine the effects of COVID-19 upon adolescents' and youth's access to health services in a humanitarian context. Given the paucity of data available on the experiences of adolescents and youth accessing SRH services in crisis settings, let alone during the most recent global crisis, this study was urgently needed to highlight the needs of adolescents and youth during COVID-19 and to help address critical gaps and barriers to service uptake. While the COVID-19 pandemic continues at the time of publishing this article, the discussion below will highlight these results in the wider context of COVID-19 and other recent pandemics and show how to adapt these proposed solutions for a continuing dynamic pandemic.

As identified in the few other studies that have looked at barriers to SRH access and use among adolescents and youth in emergency settings, poor access to adolescent- and youth-friendly information and services, including mental and psychosocial support services, was a consistent theme throughout many of the gathered stories and transcripts from the focus group discussions with adolescents and youth (26). This research highlighted how overlapping crises affect the provision of health services and other humanitarian services and programs for adolescents and youth. As noted in other literature, the onset of an emergency does not decrease SRH needs for adolescents and youth but increases those needs and jeopardizes access and use of services (7). In the case of COVID-19, this situation created new challenges to operating for health and humanitarian staff, while exposing some existing problems related to the provision of services to adolescents and youth.

Similar to the situation faced during Ebola virus disease outbreaks, health workers in Mahama camp were put in difficult positions of triaging patients while protecting others from transmission of COVID-19. Restrictions on the number of humanitarian staff permitted to the camp added additional challenges for fewer health staff to treat a growing number of patients. Other services were also adversely affected, such as youth programming and community mobilization activities, at the same time as schools were closing and lockdown and social distancing guidelines were being enforced. Fewer employment opportunities were available to households, including to adolescents and youth, due to lockdown protocols to prevent the spread of COVID-19. All these factors increased the SRH needs of adolescents and youth and negatively impacted how adolescents and youth were able to access and receive SRH information and services in Mahama camp.

While ensuring patients with fevers received timely and effective treatment, health staff were unable to meet the needs of other patients with urgent needs, such as an adolescent requesting emergency contraception within the 72 hours window. Adolescents and youth experienced significant delays and were told to come back to the health facility when doctors and nurses were unable to examine or provide consultations to adolescents and youth for general SRH questions, pregnancy and STI screening, contraceptive counseling and service provision, treatment of STIs, and treatment for menstrual cramps. Practitioners should examine how triage categories are created and ensure that access to emergency contraception, pregnancy testing, and testing and treatment for STIs is prioritized for all patients, including adolescents and youth. Practitioners should also ensure that youth coordinators or father-educators are on hand to support adolescents and youth in getting access to the appropriate services. All four focus group discussions agreed that COVID-19 triage and treatment protocols need to be re-examined to better address the concerns of adolescents and youth in a timely and respectful manner.

The economic challenges households faced as a result of COVID-19 forced many adolescents and youth to utilize negative coping mechanisms, including selling sex in exchange for goods or services. This increased their risk for unplanned pregnancy, STIs, and sexual abuse, exploitation, and violence. Practitioners understand how crises affect households' ability to pay for basic items (including soap and menstrual hygiene management supplies) and should make sure non-food items, cash, vouchers, and other financial assistance options are included in preparedness, readiness, and response plans. These plans should include employment opportunities for adolescents and youth, as they are particularly vulnerable to sexual abuse and exploitation in times of crisis.

Some of the issues that surfaced from adolescents and youth were not the result of COVID-19 but became exacerbated as human resources became more constricted. Misunderstandings of health policies and best practices resulted in service providers requiring adolescents and youth to bring their sexual partner in order to receive certain services, including pregnancy tests, STI tests and treatment, and contraceptive methods. Additional discussions with health program staff clarified that this practice is meant to help support the patient's partner with receiving testing and treatment for STIs and/or information regarding maternal and newborn health services; however, the application of this practice led adolescents and youth to believe that they could not receive services without their partners present at the facility. Input collected during focus group discussions noted this as a key policy that needs to be removed. Practitioners should review protocols surrounding SRH information and service provision to adolescents and youth, particularly surrounding parental consent and conditions for receiving care. During a pandemic crisis, all efforts should be made to reduce barriers and restrictions for adolescents and youth to receive SRH information and the full range of SRH services, regardless of age, gender, marital status, or other attributes. Practitioners and SRH advocates can be working to review these protocols and policies during COVID-19, as the situation continues to osculate and the effects of the pandemic will continue to affect Rwanda and other countries for months to come.

Preexisting attitudes and biases toward adolescents and youth receiving SRH information and services appears to have been a factor with how adolescents and youth were treated in waiting rooms and during consultations for SRH services. Helping staff understand what services are guaranteed to adolescents and youth, how their needs are different from other patients, and how to best provide services to them could help address some of the concerns raised in adolescent and youth stories. Adolescent and youth participants from the focus group discussions added that orientation and trainings on SRH information and service provision should not be constricted to only health staff but should also include CHWs and peer educators as well. The suggestions from adolescents and youth to have permanent staff dedicated to adolescents and youth at the facility and youth-friendly center would provide additional avenues to make them feel more welcome and comfortable receiving SRH services. Additionally, as recommended by adolescents and youth, humanitarian staff should ensure clients can provide feedback on their experience and the services. These accountability mechanisms should be clearly communicated to community members, explaining that all feedback provided through these channels is confidential and will be used to address identified issues. These accountability measures can be established during COVID-19 without increasing the risk of transmission *via* hotlines, feedback boxes, and other mechanisms; however, these systems should only be introduced if there is an adequate level of human resource capacity to review and discuss the concerns raised.

Adolescents and youth cited community mobilization and youth programming as critical sources of information, education, and support. Following global best practices, practitioners should consult community members and adolescents and youth on what to include in SRH messaging and how to deliver these messages. As the situation allows, these activities should continue and diversify to include other communication channels—taking advantage of football matches, theaters, and other suggested entry points by adolescents and youth, while using social distancing protocols to decrease the risk of transmission of COVID-19. The stories and focus group discussion findings from adolescents and youth, as well as discussions with community members and health providers, underscore the need to expand community mobilization efforts beyond adolescents and youth. The stigma surrounding sexual activity among adolescents and youth and those who seek SRH services following sexual activity should be discussed among all community members in the camp. More work is required to build relationships between health providers, community members, particularly influential leaders, and adolescents and youth to create trust and ensure the community understands the services the health facility is providing and why these services are necessary for adolescents and youth. As recommended by health providers and community members, organizations should also utilize teachers and other community mobilization staff, such as father-educators and CHWs, to disseminate messaging throughout the camp.

The data collected from this research provides future areas of exploration, including disaggregating the collected stories by age, gender, marital status, and other characteristics of the adolescent. Results from the SenseMaker methodology, including modulator results, could also be examined against these findings and potentially provide further understanding of the situation facing refugee adolescents and youth during COVID-19. As this methodology has been employed in a few humanitarian settings with adolescents, the findings from this study could be used together with other research studies to understand if SenseMaker is an appropriate tool for humanitarian contexts and whether it should be used with adolescents and youth users.

## Data Availability Statement

The raw data supporting the conclusions of this article will be made available by the authors, without undue reservation.

## Ethics Statement

The studies involving human participants were reviewed and approved by Rwanda National Ethics Committee. Written informed consent to participate in this study was provided by the participants' legal guardian/next of kin.

## Author Contributions

KM, MA, and JH conceived of the presented idea. KM conducted the literature review. KM and CW completed the analyses. MA and JH provided programmatic inputs. KM, CW, and MG drafted the manuscript. All authors discussed the results and discussion, and contributed to the final manuscript.

## Funding

This study was made possible with funding from the Government of Netherlands Ministry of Foreign Affairs.

## Conflict of Interest

The authors declare that the research was conducted in the absence of any commercial or financial relationships that could be construed as a potential conflict of interest.

## Publisher's Note

All claims expressed in this article are solely those of the authors and do not necessarily represent those of their affiliated organizations, or those of the publisher, the editors and the reviewers. Any product that may be evaluated in this article, or claim that may be made by its manufacturer, is not guaranteed or endorsed by the publisher.
