# Corrigendum: Understanding the Sexual and Reproductive Health Experiences of Refugee and Host Community Adolescents and Youth in Rwanda During COVID-19: Needs, Barriers, and Opportunities

**DOI:** 10.3389/frph.2022.909946

**Published:** 2022-04-20

**Authors:** Katherine Meyer, Monique Abimpaye, Jean de Dieu Harerimana, Christina Williams, Meghan C. Gallagher

**Affiliations:** ^1^Save the Children USA, Department of Global Health, Washington, DC, United States; ^2^Save the Children International, Rwanda-Burundi Country Office, Kigali, Rwanda

**Keywords:** adolescent, youth, COVID-19, sexual, reproductive, health, humanitarian, refugee

In the published article, there was an error in affiliation 1. Instead of “Department of Global Health, Washington, DC, United States,” it should be “Save the Children USA, Department of Global Health, Washington, DC, United States.” The authors apologize for this error and state that this does not change the scientific conclusions of the article in any way. The original article has been updated.

In the published article, there was an error in affiliation 2. Instead of “Save the Children International, Democratic Republic of Congo Country Office, Goma, Democratic Republic of Congo,” it should be “Save the Children International, Rwanda-Burundi Country Office, Kigali, Rwanda.” The authors apologize for this error and state that this does not change the scientific conclusions of the article in any way. The original article has been updated.

## Publisher's Note

All claims expressed in this article are solely those of the authors and do not necessarily represent those of their affiliated organizations, or those of the publisher, the editors and the reviewers. Any product that may be evaluated in this article, or claim that may be made by its manufacturer, is not guaranteed or endorsed by the publisher.

